# Interspecific Hybridization Yields Strategy for South Pacific Filariasis Vector Elimination

**DOI:** 10.1371/journal.pntd.0000129

**Published:** 2008-01-16

**Authors:** Corey L. Brelsfoard, Yves Séchan, Stephen L. Dobson

**Affiliations:** 1 Department of Entomology, University of Kentucky, Lexington, Kentucky, United States of America; 2 Institut Louis Malardé, Papeete, French Polynesia; New York University School of Medicine, United States of America

## Abstract

**Background:**

Lymphatic filariasis (LF) is a leading cause of disability in South Pacific regions, where >96% of the 1.7 million population are at risk of LF infection. As part of current global campaign, mass drug administration (MDA) has effectively reduced lymphatic filiariasis prevalence, but mosquito vector biology can complicate the MDA strategy. In some regions, there is evidence that the goal of LF elimination cannot be attained via MDA alone. Obligate vector mosquitoes provide additional targets for breaking the LF transmission cycle, but existing methods are ineffective for controlling the primary vector throughout much of the South Pacific, *Aedes polynesiensis*.

**Methodology/Principal Findings:**

Here we demonstrate that interspecific hybridization and introgression results in an *A. polynesiensis* strain (‘CP’ strain) that is stably infected with the endosymbiotic *Wolbachia* bacteria from *Aedes riversi*. The CP strain is bi-directionally incompatible with naturally infected mosquitoes, resulting in female sterility. Laboratory assays demonstrate that CP males are equally competitive, resulting in population elimination when CP males are introduced into wild type *A. polynesiensis* populations.

**Conclusions/Significance:**

The findings demonstrate strategy feasibility and encourage field tests of the vector elimination strategy as a supplement to ongoing MDA efforts.

## Author Summary

Lymphatic filariasis (LF) is a global health problem, with over 120 million people affected annually. The current LF elimination program is focused on administering anti-filarial drugs to the entire at-risk population via annual mass drug administration (MDA). While the MDA program is proving effective in many areas, other areas may require augmentative measures such as vector control. An example of the latter is provided by some regions of the South Pacific where *Aedes polynesiensis* is the primary vector. Here, we describe a novel vector control approach based upon naturally occurring *Wolbachia* bacterial infections. *Wolbachia* are endosymbiotic intracellular bacteria that cause a form of sterility known as cytoplasmic incompatibility. We show that introgression crosses with mosquitoes that are infected with a different *Wolbachia* type results in an *A. polynesiensis* strain (designated ‘CP’) that is incompatible with naturally infected mosquitoes. No difference in mating competitiveness is observed between CP males and wild type males in laboratory assays. The results support continued development of the strategy as a tool to improve public health.

## Introduction

Lymphatic filariasis (LF) is a global health problem, with over 120 million infected individuals and an estimated one billion people at risk of infection [1]. The current LF elimination campaign is premised upon the lack of a non-human reservoir for *Wuchereria bancrofti* and is enabled by recent advances in diagnostic tools and treatment as well as the donation of microfilaricidal drugs [1]–[3]. In the absence of appropriate macrofilaricidal prophylactic or therapeutic treatments, the current strategy focuses on interruption of LF transmission via Mass Drug Administration (MDA): treatment of the entire ‘at risk’ population with microfilaricidal compounds to suppress microfilariae levels below that required to sustain transmission. The MDA strategy calls for drug treatment to continue annually over a period exceeding the ∼5 year lifespan of adult worms, [4] with a goal of global LF elimination by 2020.

The efficacy of the MDA strategy is compromised in some regions by the biology of the insect vectors. A notable example is provided in endemic areas within the South Pacific, where the diurnal subperiodic form of *W. bancrofti* is transmitted by *A. polynesiensis*. *A. polynesiensis* displays a pattern of negative density dependent transmission, such that this mosquito is a more efficient vector in low-level microfilaraemics, such as that which occurs with MDA strategies [5],[6]. The stabilizing impact of negative density dependent transmission is hypothesized as a contributor to an inability to eliminate LF in French Polynesia despite decades of ongoing MDA [5],[7]. Since mosquitoes are an obligate host for *W. bancrofti*, anti-vector interventions are recognized as a supplemental method to break the LF transmission cycle, leading to recommendations for the integration of vector control with MDA in areas where *A. polynesiensis* is the primary vector [3]. Unfortunately, effective control of *A. polynesiensis* has never been accomplished, due to problems including the inaccessibility of *A. polynesiensis* breeding sites and geography of Pacific island nations, which complicate ongoing vector control efforts [3],[8]. In contrast, Pacific island geography can simplify and facilitate an area-wide elimination approach, by subdividing vector mosquitoes into discrete populations with limited immigration.

Area-wide elimination programs targeting mosquito populations have been attempted previously, with mixed results [9]. A notable success was reported in a field trial in which a *Culex quinquefasciatus* population was eliminated via repeated, inundative releases of male mosquitoes infected with an incompatible *Wolbachia pipientis* bacteria [10]. *Wolbachia* are obligate intracellular bacteria that are maternally inherited in insects and other invertebrates [11]. In mosquitoes, *Wolbachia* cause a form of sterility known as cytoplasmic incompatibility (CI), which results in karyogamy failure and arrested embryonic development. In populations where individuals are infected with different *Wolbachia* types, bi-directional CI can occur: sterility results in both cross directions between mates infected with different *Wolbachia* types. Models predict that in natural populations, sterility resulting from bi-directional CI is a transient event, since one infection will predominate and replace the other cytotype [12]. In the *Wolbachia*-based vector control strategy however, female sterility is artificially sustained by repeated, inundative releases of incompatible males analogous to traditional sterile insect technique (SIT) [13], resulting in mosquito population decrease and elimination. It is emphasized that the released male mosquitoes do not blood feed, vector disease or transmit *Wolbachia*. Further development and expansion of the *Wolbachia*-based suppression approach was not subsequently pursued due to strategy complications including immigration of mated females and variable CI patterns observed in *Culex* populations in different geographic areas. Furthermore, the application was viewed as specialized to *Culex*, since bi-directional CI was not observed in additional vector species. The latter problem has recently been addressed with a demonstrated ability to artificially generate new *Wolbachia* infection types in mosquitoes [14].

Problems with insecticidal approaches (e.g., resistance and non-target effects) have led to a renaissance of interest in genetic control of disease vectors, using newly-developed transgenic approaches such as the Repressible Dominant Lethal (RIDL) technology [15]. With the new reality of potential transgenic insect releases, considerable thought is being devoted to addressing the requirements for field implementation of an approach employing transgenic mosquitoes. It is recognized that experience is lacking that demonstrates the efficacy and safety of the transgenic mosquito approach, and a critical question relates to the social acceptance toward the release of transgenic mosquitoes without a demonstrated benefit (i.e., an epidemiologically significant impact on transmission of mosquito borne disease) to offset real and perceived risks. Here we describe progress toward a non-transgenic approach for primary vector elimination within an endemic area, which can allow the subsequent evaluation for an epidemiological impact on disease transmission.

## Methods

### Insect rearing and crosses

**Figure 1:**
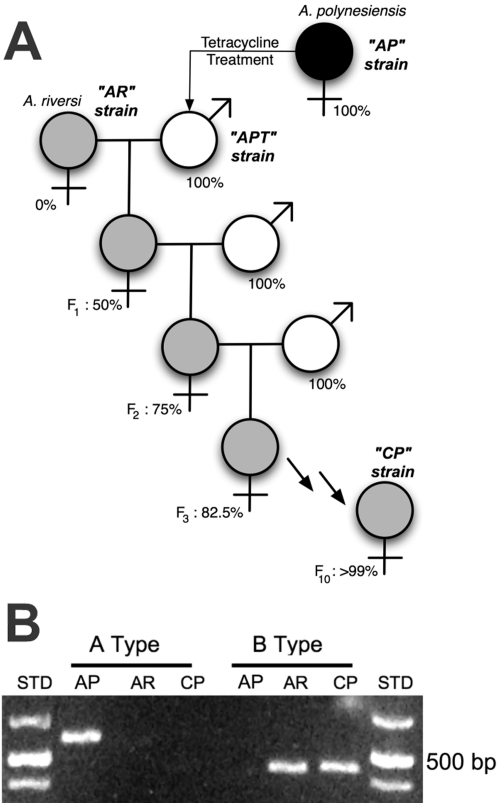
Introgression Strategy and PCR confirmation of *Wolbachia* infection type. (A) *Wolbachia* infection type is indicated by symbol shading: gray-filled symbols represent the B type *Wolbachia* infection from *A. riversi*; the black-filled symbol represents the A type *Wolbachia* infection from *A. polynesiensis* (AP strain); unshaded symbols represent aposymbiotic individuals (APT strain). The theoretical percentage of *A. polynesiensis* genotype is shown as a percentage below symbols. The *Wolbachia* infection is maternally inherited, while the genotype is inherited from both parents. Repeated introgression of hybrid females with APT males results in the CP strain. (B) *Wolbachia* type-specific primers demonstrate that the CP strain infection type is the same as that of the original *A. riversi* strain (AR) and differs from the infection that naturally occurs in *A. polynesiensis* (AP). *Wolbachia* type-specific primers used in amplification reactions are indicated above the horizontal lines. STD is the molecular weight marker: 1kb DNA Ladder Plus (Fermentas Inc., Hanover, MD).

Mosquito strains and details of maintenance and experimental crosses are as previously described [16]: AR = *Aedes riversi*; ART = Aposymbiotic AR strain (*Wolbachia* removed via tetracycline treatment); AP = *Aedes polynesiensis*; APT = Aposymbiotic AP. Each generation of introgression (Figure 1A) was established with >100 individuals of each sex.

For the CP male competitiveness assay, 25 cages were established, each with ten virgin *A. polynesiensis* females (<2 days post eclosion) and 20 males (<3 days post eclosion). Five days after introduction, females were blood fed, isolated and allowed to oviposit. The egg hatch assay consisted of allowing one week for egg maturation, submerging eggs for three days, and then observing eggs from individual females using a dissection scope. Females producing fewer than ten eggs were excluded from the data set. To confirm female insemination, spermathecae were checked for females producing broods with low egg hatch.

### Microsatellite analysis

DNA was extracted from individual mosquitoes using DNeasy kits (Qiagen, Valencia, CA) following manufacturers instructions. Six-microsatellite primer pairs were used to amplify loci using PCR conditions as previously described [17]. Left primers were fluorescent labelled with different WellRED dye colors (Integrated DNA technologies, Coralville, IA). Fragments sizes were measured using a CEQ 2000 sequencer (Beckman Coulter, Fullerton, CA) according to manufacturer's instructions. Allele frequencies and genotypic dis-equilibrium were calculated, and Fisher's exact tests [18] were performed using GENEPOP version 3.4. All calculations were performed using the Markov chain method with demorization set to 1000, 100 batches, and 1000 iterations per batch.

### DNA extraction, PCR, and maternal inheritance assay

Adults were homogenized in 100 µl of buffer containing 10 mM Tris-HCL, 1 mM EDTA, and 50 mM NaCl, at pH 8.2 using a Mini-beadbeater (BioSpec Products, Inc., Bartlesville, OK). After homogenization, samples were boiled for 5 min and centrifuged at 16,000*g* for 5 min. One µl of supernatant was used for each PCR reaction. PCR conditions were as described previously [19]. Infection type of CP, AP, and AR was determined using PCR primers specific for A type *Wolbachia* (136F and 691R) or B type *Wolbachia* (81F and 522R) [20]. To assess maternal inheritance rates, CP females were mated with CP males, blood fed, isolated, and allowed to oviposit. CP females and their progeny were examined via PCR using the 81F and 691R primers [20] using the above-described methods.

### Statistical analysis

To analyze male mating competitiveness a Chi-square goodness of fit test was performed to compare observed and expected numbers of hatching broods for the replicate cages of the varying ratios of CP:AP males. To analyze population suppression in replicate cages, multiple Mann-Whitney tests, with sequential Bonferroni correction were conducted to compare egg hatch rates between cages with varying ratios of CP:AP males. Compatible female egg hatch data from all replicate cages was subjected to a Kruskal-Wallis test.

## Results

Prior studies demonstrate that *A. polynesiensis* and *A. riversi,* two closely related members of the *Aedes* (*Stegomyia*) *scutellaris* complex are naturally infected with differing *Wolbachia* infection types (A and B clades, respectively) [16], and that removal of the *Wolbachia* infection results in egg hatch, which does not occur in interspecific crosses of naturally-infected individuals. Here, we report that hybrids resulting from crosses of uninfected (aposymbiotic) *A. polynesiensis* and *A. riversi* are viable and fertile (Table 1). Hybrid fertility allows a strategy in which the *A. riversi Wolbachia* type is introgressed into the *A. polynesiensis* genotype, resulting in the ‘CP’ strain (Figure 1A). As shown in Figure 1B, PCR confirms that *Wolbachia* in the CP strain is B-type *Wolbachia,* as predicted.

**Table 1:** Crosses and Pattern of Cytoplasmic Incompatibility

Female x Male			Percent Egg Hatch±s.e.m.; number of cross replicates
AR	x	APT a	13.6±17%; *n* = 14
ART	x	APT b	27.8±26.6%; *n* = 14
AP	x	ART	0.2±0.5%; *n* = 7
APT	x	ART	0.6±1.8%; *n* = 21
APT	x	CPT	59.1±43.5%; *n* = 7
CPT	x	APT	73.7±23.4%; *n* = 7
CP	x	AP	0.23±0.11%; *n* = 18
AP	x	AP	87.8±9.7%; *n* = 8
AP	x	CP	0.0±0%; *n* = 28
CP	x	CP	62.1±4.01%; *n* = 18

AR = *Aedes riversi*; ART = Aposymbiotic AR strain; AP = *Aedes polynesiensis*; APT = Aposymbiotic AP

a Hybrid progeny designated as “CP line” (see Figure 1a)

b Hybrid progeny designated as “CPT line”

**Figure 2:**
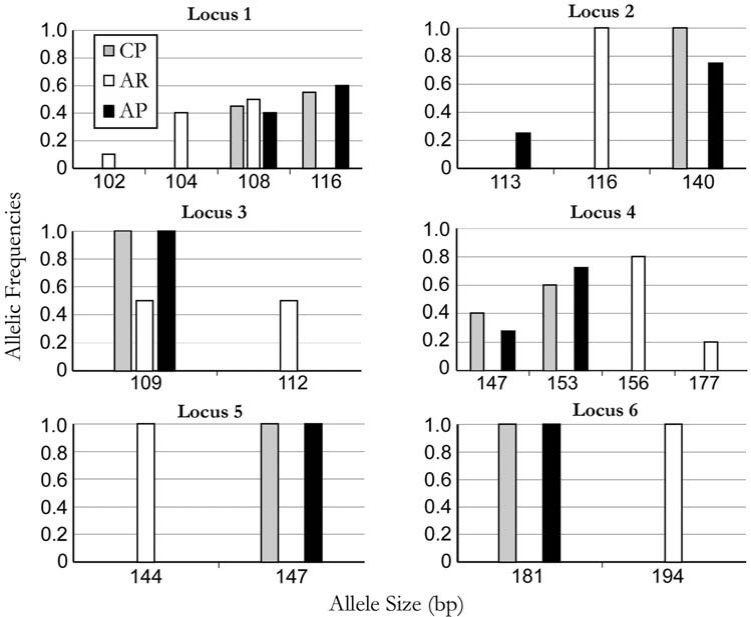
Microsatellite allelic frequencies. Panels represent the allelic frequencies of the six microsatellite loci (*Loci 1-6*), which are examined for each mosquito strain (AP, AR and CP). In each panel, the Y-axis denotes allelic frequencies and the x-axis denotes the size of each fragment in base pairs. *Loci* 1-6 correspond to previously reported microsatellite loci AP1-AP6 [17] (naming modified here to avoid ambiguity with mosquito strains).

To examine introgression of the CP strain, allelic distributions of six-microsatellite loci were compared between the AP, AR and CP strains. All loci investigated are polymorphic between strains, and four loci are polymorphic within strains. The test for genotypic dis-equilibrium across all pairs was not significant (p>0.2), suggesting loci are not linked. The CP and AP strains were observed to share a similar distribution of alleles across all loci (Fisher's exact test; P>0.3). In contrast, allele distributions of CP and AP are significantly different from AR (Fisher's exact test, P<0.0001), with only two alleles commonly shared by all three strains at two loci (Loci 1 and 3) (Figure 2). Thus, the results are consistent with the hypothesized CP introgression with the AP genotype.

Crosses demonstrate strong bi-directional incompatibility between CP and naturally infected *A. polynesiensis*, with no egg hatch resulting from >1,800 eggs examined in crosses of AP females and CP males (Table 1). Progeny resulting from crosses of aposymbiotic CPT males (Table 1) demonstrates that the observed sterility in CP crosses is due to the *Wolbachia* infection.

Experiments show a high level (>99% fidelity) of maternal transmission of *Wolbachia* from CP females to both sons and daughters. Subsequent to oviposition, CP females were confirmed to be infected using PCR with *Wolbachia*-specific primers. Approximately ten daughters and ten sons from each of ten infected CP females were PCR tested, and all were observed to be *Wolbachia* infected (n = 210). The high maternal inheritance rate is similar to that observed in naturally-infected *A. polynesiensis* and a related mosquito: *Aedes albopictus* [19],[21].

**Figure 3:**
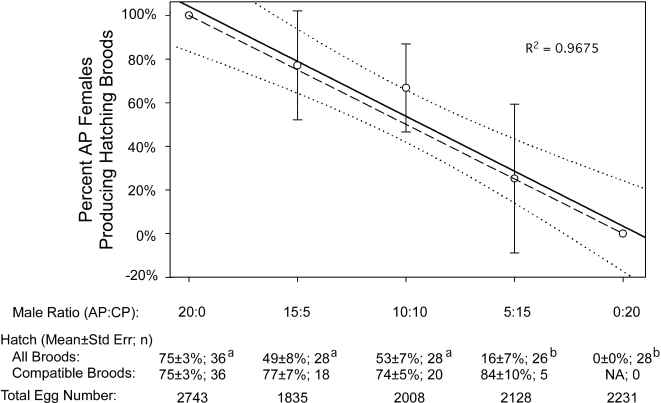
*A. polynesiensis* suppression strategy and CP male competitiveness. Based upon the observed egg hatch rate, females are scored as either ‘compatible mating’ ( = eggs hatching) or ‘incompatible mating’ ( = eggs not hatching). The percent compatible females (compatible females/total females) is determined for each cage replicate (10 females/cage; 5 cage replicates/treatment). Circles and bars indicate the mean±standard deviation for each treatment (i.e., male ratio). The trend line (solid line) with 95% confidence intervals (dotted lines) are generated based upon the observed values. Predicted values (dashed line) are calculated assuming equal competitiveness of CP and AP males. Below the graph, egg hatch rates are based upon the combined oviposition of females within cages. Differing superscripted letters indicate significant differences (Two-tailed, Mann-Whitney, P<0.01, Bonferroni corrected). R^2^ value is shown for the trend line fitted to observations.

Strong bi-directional CI and high maternal transmission support investigation of a vector control strategy in which CP male releases are used to suppress *A. polynesiensis* populations. To examine the strategy: virgin AP females were introduced into cages with varying ratios of CP:AP males (Figure 3). Following mating, females were isolated and the egg hatch rate was examined. As shown in Figure 3, the egg hatch was observed to significantly decrease from 75% to 0% egg hatch, inversely related to the frequency of incompatible CP males. The experimental design also permits an assessment of CP male competitiveness relative to AP males. As illustrated in Figure 3, the number of observed compatibly mated females (i.e., producing hatching broods) did not differ from predictions that assume equal male competitiveness (Chi Square; P>0.1). The data shown in Figure 3 also support the hypothesis that females utilize sperm from one male. A comparison of egg hatch rates resulting from compatibly-mated females did not differ significantly between treatments (Kruskal-Wallis, P>0.3). If females were to utilize sperm from multiple males, then a lower egg hatch rate would be expected in treatments with a mixture of CP and AP males. The results support the feasibility of a CI-based *A. polynesiensis* suppression strategy and encourage additional experiments to assess the strategy under more natural conditions (e.g., field cages).

## Discussion

The inability of prior MDA efforts to eliminate LF transmission from some Pacific regions represents a potential weakness in the current global campaign. Therefore, an ability to reduce or eliminate the required mosquito vector populations would provide a useful augmentative tool for blocking LF transmission. *A. polynesiensis* populations provide a logical target, given their broad geographic range in the South Pacific and ability to vector filariasis in low-level microfilaraemics (i.e., ‘limitation’ LF transmission) [5],[7]. Unfortunately, existing vector control tools have proven unsuccessful against this mosquito species.

The results presented here support the feasibility of a species-specific approach in which inundative releases of bi-directionally incompatible males induce sterility in *A. polynesiensis* females, resulting in vector population elimination. The geography of the South Pacific is ideal for the proposed *A. polynesiensis* suppression strategy, since the *A. polynesiensis* population is subdivided into islands with limited immigration [22],[23]. The natural subdivision of *A. polynesiensis* into isolated populations will facilitate a sequential elimination approach, in which transient entomological teams focus effort on one island and then progress to a subsequent island. Following elimination, a reporting system would be deployed, monitoring for *A. polynesiensis* reintroduction and reestablishment.

The proposed strategy would be integrated with the existing MDA strategy, to be deployed in areas where LF elimination is complicated by *A. polynesiensis* biology. It is emphasized that the primary goal of breaking the LF transmission cycle does not require the permanent eradication of *A. polynesiensis*. Instead, a transient elimination of *A. polynesiensis* will suffice, as long as the period of vector elimination extends beyond the lifespan of adult *W. bancrofti* in the reservoir human population. However, *A. polynesiensis* eradication would be desirable from the broader public health perspective that *A. polynesiensis* is a biting nuisance and serves as a vector during periodic dengue epidemics. The successful demonstration that releases of bi-directionally incompatible males can impact LF transmission will encourage an extension of the strategy to a broader geographic range and to additional vector species (e.g., *Culex* spp. or other aedine LF vectors in the South Pacific) using the previously demonstrated ability to artificially generate novel *Wolbachia* infections in medically important mosquitoes [14].

A concern relates to downstream logistical aspects associated with the subsequent ‘scale up’ required for suppression of larger populations. Due to bi-directional CI, females that are unintentionally released are incompatible with wild type males. With the reduction of the population size due to CI-induced sterility, there is an increasing probability that accidental female releases will permit the establishment of the new infection type, resulting in population replacement instead of population elimination [12]. Thus, strategy success requires releases to consist of males only. While a variety of mechanical sex separation tools for mosquitoes have been developed, available devices are not sufficiently accurate. Therefore, to ensure male-only releases, early field trials will be on a relatively small scale that allows visual verification of mechanically-separated males, similar to prior trials [10]. In the event that visual inspection is not cost effective for subsequent large-scale releases, deployment of the proposed approach in larger areas may require additional technology to improve cost efficacy, such as genetic sexing [24]. An additional possibility is premised upon the observation that female mosquitoes are typically more susceptible to radiation relative to males [25] and would treat release individuals with low levels of radiation to render unintentionally released females impotent (i.e., sterile or of negligible fitness). The risk of compatible matings between released CP males and *A. riversi* females in the field is not a concern, since populations of *A. riversi* occur in Japan, [26] remote from South Pacific islands that are proposed for field releases.

The non-transgenic, species-specific, *Wolbachia*-based elimination strategy proposed here provides a logical segue toward transgenic approaches (i.e., RIDL [15]), which may yield improved efficacy and/or cost. Furthermore, the social palatability of transgenic mosquito releases can be increased via an approach that is integrated with *Wolbachia*-induced CI. Specifically, if released transgenic males are cytoplasmically incompatible with the targeted mosquito population, the released transgene has a reduced probability of establishing in the field.

Future efforts must define the vectorial competency of CP females relative to wild-type females. In the event that the CP strain is observed to be refractory to LF transmission, replacement of the naturally-susceptible wild-type population with a refractory CP population may be a desirable outcome. Based upon the results of prior vector competency studies examining hybridizations between members of the *Aedes scutellaris* complex, the prospect of CP displaying reduced vectorial competency is not a remote possibility. Notably: prior cross experiments demonstrate that the *Wuchereria* refractoriness phenotype is dominant, [27],[28] prior hybridization experiments provide evidence for cytoplasmic inheritance of susceptibility [29] and AR is not recognized as a disease vector. Since periodic dengue epidemics occur within the *A. polynesiensis* range, the competency of CP to transmit dengue and additional pathogens (e.g., chikungunya) would need to be assessed prior to implementing a population replacement program. The latter complication would be less of a concern with an elimination strategy, since following successful intervention, neither CP or *A. polynesiensis* would occur in the field.

## Supporting Information

Alternative Language Abstract S1 Abstract translated into French.
(0.02 MB DOC)

Alternative Language Abstract S2 Abstract translated into Spanish.
(0.02 MB DOC)
