# Supplementary material for: Interspecific Hybridization Yields Strategy for South Pacific Filariasis Vector Elimination
Source: PLoS Negl Trop Dis. 2008 Jan 16;2(1):e129. doi: 10.1371/journal.pntd.0000129 (PMC2217672; doi:10.1371/journal.pntd.0000129)
Supplement: Alternative Language Abstract S1 — Abstract translated into French. (0.02 MB DOC) [file pntd.0000129.s001.doc]

**Abstract**

**Introduction.** La filariose lymphatique menace près de 96% des 1,7 millions d’habitants du Pacifique Sud et représente de fait l’une des principales causes de morbidité dans cette région du monde. Grace à la campagne globale d’éradication, le traitement thérapeutique annuel a permis de réduire de façon significative la prévalence de cette maladie. Cependant, la biologie du moustique vecteur affecte l’impact de cette sratégie unique. Dans certaines régions il est maintenant clair que le traitement thérapeutique seul ne permettra pas d’éliminer la filariose lymphatique. Vecteurs obligatoires, les moustiques constituent une cible supplémentaire permettant de rompre le cycle de transmission de la maladie. Les méthodes conventionnelles actuelles sont cependant inadaptées dans la lutte contre *Aedes polynesiensis*, le vecteur principal de la filariose lymphatique dans la Pacifique Sud.

**Méthodologie/Résultats principaux**

Nous démontrons ici qu’ hybridation interspécifique et introgression permettent de générer une souche d’*A. polynesiensis* (souche ‘CP’) infectée de façon stable avec l’endosymbiote bactérien *Wolbachia* d’*Aedes riversi*. La souche CP incompatible de façon bi-directionnelle avec les moustiques infectés naturellement, induit la stérilité chez les femelles. Des tests en laboratoires démontrent que les mâles CP sont aussi compétitifs, conduisant à l’élimination d’une population lorsque ceux ci sont introduit dans des populations sauvages d’*A. polynesiensis*.

**Conclusions/Importance.** Ces observations démontrent la faisabilité de la stratégie d’élimination vectorielle et encouragent la réalisation de tests sur le terrain en complément des efforts de traitement thérapeutique actuels.

French translation of the abstract by Hervé Bossin (Institut Louis Malardé, Papeete, French Polynesia)
