# Supplementary material for: Interspecific Hybridization Yields Strategy for South Pacific Filariasis Vector Elimination
Source: PLoS Negl Trop Dis. 2008 Jan 16;2(1):e129. doi: 10.1371/journal.pntd.0000129 (PMC2217672; doi:10.1371/journal.pntd.0000129)
Supplement: Alternative Language Abstract S2 — Abstract translated into Spanish. (0.02 MB DOC) [file pntd.0000129.s002.doc]

**MS#** **07-PNTD-RA-0110R2**

**Abstract - Spanish Translation**

*Translation Credit:*

Gary N. Fritz

Eastern Illinois University

Biological Sciences Dept.

Life Science Bldg. 2070

600 Lincoln Avenue

Charleston, IL 61920

Phone# 217-581-2514

Resumen

**Introducción:** En el Pacifico del Sur, la filariasis limfatica (FL) afecta mas del 96% de los 1.7 milliones de habitants en riesgo, Y es una de las causas mas importantes de incapacidad. Aunque la Administración de Drogas en Masa (ADM), como parte de la campaña global actualizada, ha sido efectivo en la reducción de la prevalencia de la FL, la biologia del vector (mosquito) puede complicar la estrategia del ADM. En algunas regiones, hay evidencia indicativo que la eliminación de la FL no se logrará unicamente via la ADM. Otra oportunidad para interrumpir el ciclo de transmisión existe mediante el enfoque en los vectores obligatorios de la FL, pero los metodos existentes son inefectivos en tanto al control del vector primario mediante mucha de la region del Pacifico del Sur: *Aedes polynesiensis*.

**Metodología/Descubrimientos Primarios.** Aqui demostramos que la hibridación inter-específico y la introgreción resulta en una cepa de *A. polynesiensis* (cepa ‘CP’) infectada con la bacteria endosimbiótico *Wolbachia* de *Aedes riversi*. La cepa CP es incompatible con mosquitos silvestres e infectados, resultando en hembras esteriles. Resultados en laboratorio demuestran que la introducción de CP machos a jaulas con poblaciónes de mosquitos silvestres de *A. polynesiensis* resulta en la eliminacion de mosquitos por completo.

**Conclusiones/Significáncia.** Los resultados demuestran que esta estratégia es factible e indican la necesidad de estúdios en campo sobre la eliminación de vectores como estratégia complementaria con el ADM.
